# Meleagrin, a New FabI Inhibitor from *Penicillium chryosogenum* with at Least One Additional Mode of Action

**DOI:** 10.1371/journal.pone.0078922

**Published:** 2013-11-28

**Authors:** Chang Ji Zheng, Mi-Jin Sohn, Sangku Lee, Won-Gon Kim

**Affiliations:** Superbacteria Research Center, Korea Research Institute of Bioscience and Biotechnology, Yusong, Daejeon, Republic of Korea; University of Rochester, United States of America

## Abstract

Bacterial enoyl-acyl carrier protein reductase (FabI) is a promising novel antibacterial target. We isolated a new class of FabI inhibitor from *Penicillium chrysogenum*, which produces various antibiotics, the mechanisms of some of them are unknown. The isolated FabI inhibitor was determined to be meleagrin by mass spectroscopy and nuclear magnetic resonance spectral analyses, and its more active and inactive derivatives were chemically prepared. Consistent with their selective inhibition of *Staphylococcus aureus* FabI, meleagrin and its more active derivatives directly bound to *S. aureus* FabI in a fluorescence quenching assay, inhibited intracellular fatty acid biosynthesis and growth of *S. aureus*, and increased the minimum inhibitory concentration for *fabI*-overexpressing *S. aureus*. The compounds that were not effective against the FabK isoform, however, inhibited the growth of *Streptococcus pneumoniae* that contained only the FabK isoform. Additionally no resistant mutant to the compounds was obtained. Importantly, *fabK*-overexpressing *Escherichia coli* was not resistant to these compounds, but was resistant to triclosan. These results demonstrate that the compounds inhibited another target in addition to FabI. Thus, meleagrin is a new class of FabI inhibitor with at least one additional mode of action that could have potential for treating multidrug-resistant bacteria.

## Introduction

Multidrug-resistant bacteria such as methicillin-resistant *Staphylococcus aureus* (MRSA), vancomycin-resistant *Enterococci*, and vancomycin-resistant *S. aureus* have become an important global health concern [1], [2]. One approach to combat antibiotic resistance is to identify new drugs that can function through novel mechanisms of action. One such target is bacterial type 2 fatty acid synthesis (FASII), which is essential for bacterial cell growth [3]–[5].

FASII is conducted by a set of individual enzymes, whereas mammalian fatty acid synthesis is mediated by a single multifunctional enzyme-acyl carrier protein (ACP) complex referred to as type I. Enoyl-ACP reductase catalyzes the final and rate-limiting step of the chain-elongation process of the FASII. Four isoforms have been reported for enoyl-ACP reductase. FabI is highly conserved among most bacteria, including *S. aureus* and *Escherichia coli*. *Streptococcus pneumoniae* contains only FabK, whereas *Enterococcus faecalis* and *Pseudomonas aeruginosa* contain both FabI and FabK, and *Bacillus subtilis* contains both FabI and FabL. Recently, the FabV isoform was isolated from *Vibrio cholera*, *Pseudomonas aeruginosa*, and *Burkholderia mallei* [6], [7]. No analogue protein is present in mammals for similar transformation; thus, FabI inhibitors should not interfere with mammalian fatty acid synthesis. Because of these properties, FabI is an attractive target for antibacterial drug development [8], [9]. As drugs with single targets such as rifampicin and fosfomycin are particularly vulnerable to mutational resistance [10], FabI-specific inhibitors also have a tendency to develop resistance in bacteria by mutations that alter the drug-binding site. FabI is known to be the main target for triclosan and isoniazid, which have been used in consumer products and for treating tuberculosis, respectively [11], [12]. Triclosan-resistant bacteria and isoniazid-resistant *M. tuberculosis* are highly prevalent because of point mutations in their FabI genes [13]–[15]. In addition, rapid mutation development has been often reported in synthetic FabI inhibitors [16]. Thus, it has been recently emphasized that ideal antibiotics should bind to multiple targets [17].

Many FabI inhibitors have been reported from high-throughput screening of existing compound libraries. However, most are not suitable for the development of new antibiotics because of their lack of permeability into cell membranes and efflux in addition to their high mutational frequency [18]. The problem with such screening results lies in the compound libraries, which are systematically biased. Microorganisms produce diverse antibiotics that function in an antagonistic capacity in nature where they have competition. Most antibacterial agents in clinical use today are either microbial products or analogs [19]. A few FabI inhibitors have been reported from microorganisms [20]–[22], and most of these are phenolic compounds. Therefore, more unique FabI inhibitors need to be obtained from microorganisms.

**Figure 1:**
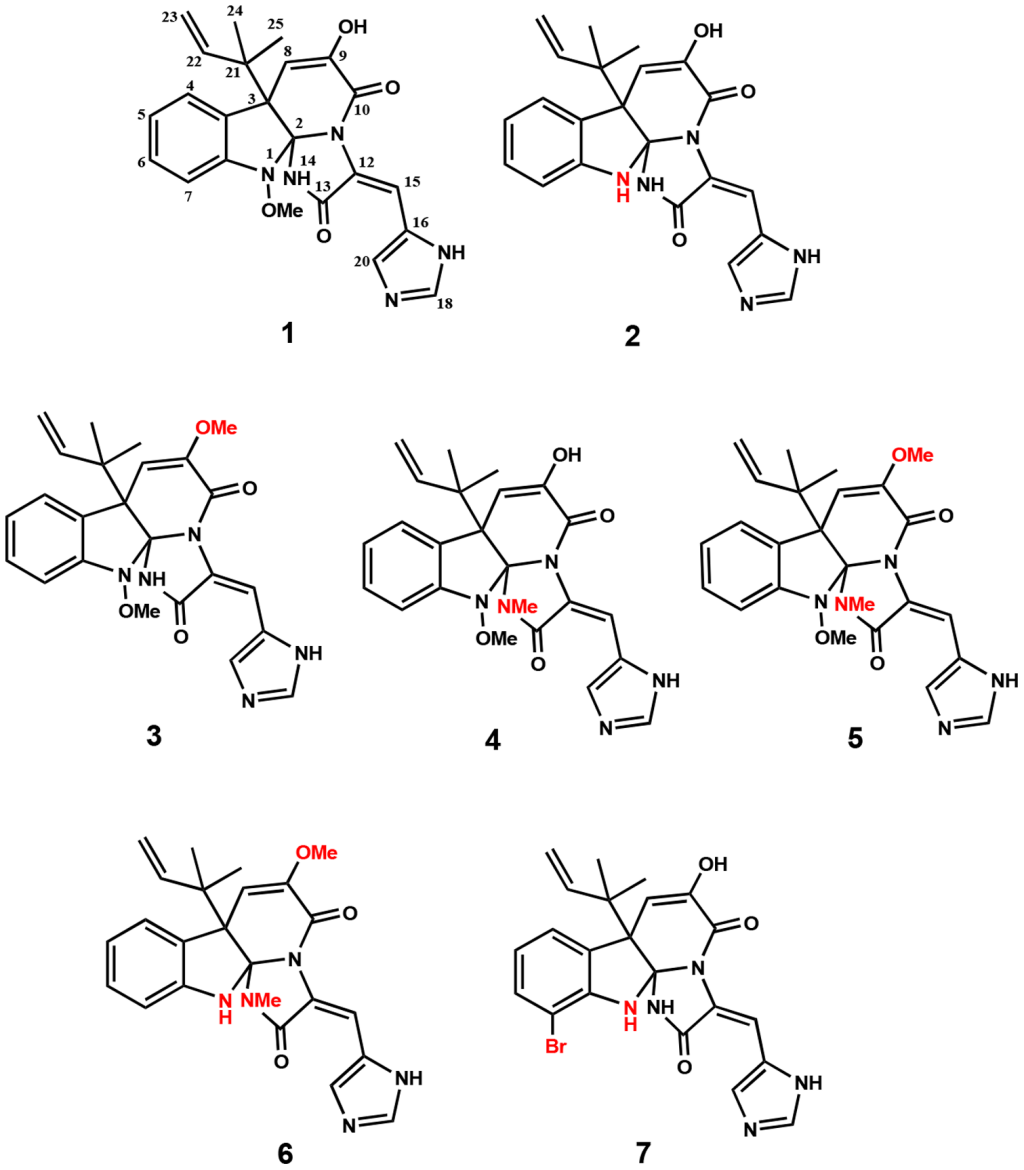
Meleagrin (1) and its chemically prepared derivatives.

During our continued screening for FabI inhibitors from microbial metabolites, we found meleagrin (**1**) with a druggable structure during solid-state fermentation of a seashore slime-derived *Penicillium chrysogenum*, a penicillin-producing species (Figure 1). Here, we report the isolation and analog preparation of meleagrin, in addition to its inhibition of FabI isoforms and whole cells of various pathogenic bacteria, target validation, and its multitarget effect.

## Materials and Methods

### Bacterial strains

The bacterial strains used in the antibacterial activity assays were obtained from the Culture Collection of Antimicrobial Resistant Microbes of Korea and the Korean Collection for Type Cultures. The pump-negative (tolC) *E. coli* EW1b was obtained from the *E. coli* Genetic Stock Center of Yale University.

### Screening and isolation of compound 1

Over 25,000 microbial extracts composed of actinomycetes and fungi were screened against *S. aureus* FabI and confirmed through a target-based whole cell assay by using *fabI*-overexpressing *S. aureus*. This analysis led to the identification of compound **1** from fungal strain F717 (Fig. 1). Compound **1** was isolated from the fermented whole medium of the fungal strain F717, which was isolated from seashore slime collected at Daechun beach, Chungcheongnam-do, Korea. The strain was identified as *Penicillium chrysogenum* based on standard biological and physiological tests and taxonomic determination. Seed culture was conducted in a liquid culture medium containing 2% glucose, 0.2% yeast extract, 0.5% peptone, 0.05% MgSO_4_, and 0.1% KH_2_PO_4_ (pH 5.7 before sterilization). A sample of the strain from a mature plate culture was inoculated into a 500-mL Erlenmeyer flask containing 80 mL of the above sterile seed liquid medium and cultured on a rotary shaker (150 rpm) at 28°C for 3 days. Subsequently, 5 mL of the seed culture was transferred into 500-mL Erlenmeyer flasks (54 flasks) containing 80 g of bran medium, which was cultivated for 7 days at 28°C to produce the active compound. The culture solid state was extracted with 80% acetone, and the extract was concentrated *in vacuo* to an aqueous solution. The aqueous solution was then extracted 3 times with an equal volume of ethyl acetate (EtOAc). The EtOAc extract was concentrated *in vacuo* to dryness. The crude extract was subjected to SiO_2_ (Merck Art No. 7734.9025) column chromatography followed by stepwise elution with CHCl_3_-MeOH (100∶1, 50∶1, and 10∶1). The active fractions eluted with CHCl_3_-MeOH (50∶1) were pooled and concentrated *in vacuo* to give an oily residue. The residue was applied again to a Sephadex LH-20 and then eluted with CHCl_3_-MeOH (1∶1). The active fraction was dissolved in MeOH and was further purified by reverse-phase high-performance liquid chromatography (20×150 mm; YMC C_18_) by using a photodiode array detector. The column was eluted using MeOH: H_2_O (75∶25) at a flow rate of 5 mL/min to afford compound **1** with >99% purity at a retention time of 19.4 min. The chemical structure of compound **1** was determined to be meleagrin [23] by mass spectroscopy (MS) and nuclear magnetic resonance (NMR) spectra as follows: [α]_D_  = −96.7° (*c* = 0.04, MeOH); HRESI-MS: *m/z* 434.18463 (M+H)^+^, C_23_H_23_N_5_O_4_ requires 434.18228; ^1^H-NMR (600 MHz, DMSO-*d_6_*): 8.30 (1H, s, NH-19), 8.17 (1H, s, H-15), 7.77 (1H, s, H-20), 7.53 (1H, d, *J* = 7.5, H-4), 7.34 (1H, s, H-18), 7.25 (1H, t, *J* = 7.5, H-6), 7.03 (1H, t, *J* = 7.5, H-5), 6.96 (1H, d, *J* = 7.5, H-7), 6.00 (1H, brs, H-22), 5.25 (1H, s, H-8), 5.01 (1H, d, *J* = 17.1, Ha-23), 4.98 (1H, d, *J* = 9.0, Hb-23), 3.66 (3H, s, 1-OCH_3_), 1.19 (6H, s, CH_3_-24 and 25), ^13^C-NMR (150 MHz, DMSO-*d_6_*): 165.0 (C-13), 158.6 (C-10), 146.2 (C-7a), 143.3 (C-22), 142.7 (C-9), 137.6 (C-20), 134.1 (C-18), 127.8 (C-6), 126.0 (C-3a), 125.9 (C-16), 124.7 (C-4), 123.7 (C-12), 123.1 (C-5), 112.8 (C-23), 111.6 (C-7), 109.2 (C-8), 106.7 (C-15), 101.5 (C-2), 64.8 (1-OCH_3_), 52.2 (C-3), 41.8 (C-21), and 23.0 (C-24 and 25).

### Preparation of derivatives of compound 1

Several derivatives of **1** were obtained by chemical modification of functional groups such as hydroxyl and amine groups (Fig. 1). Demethoxylation of compound **1** afforded glandicolin A (**2**) together with compound **7** as a byproduct. Methylation of compound **1** produced oxaline (**3**), *N^14^*-methylmeleagrin (**4**), and *O*,*N^14^*-dimethylmeleagrin (**5**). *O*,*N^14^*-dimethylglandicolin (**6**) was obtained by methylation of compound **2**. Details regarding the preparation procedures and spectral data of compounds **2–7** are presented in Information S1.

### FabI and FabK assay

*S. aureus* FabI and *E. coli* FabI enzymes were cloned, overexpressed, and purified as described previously [24]. The wild-type *fab*K gene was amplified by PCR from genomic DNA obtained from *Streptococcus pneumoniae* KCTC 5412 by using the primers 5′-GGAAACCATATGAAAACGCGTATTACGAA-3′ and 5′-CCGCTCGAGGTCATTTCTTACAACTCCTGT-3′, which contained *Nde*I and *Xho*I restriction sites, respectively. After the DNA sequence was confirmed, the gene was cloned into the pET22b vector (Novagen, Gibbstown, NJ, USA). The construct was transformed into *E. coli* BL21 (DE3) for expression following induction with isopropylthiogalactoside. The C-terminal His-tagged protein was purified as described previously [24]. Assays were conducted in half-area, 96-well microtiter plates. The compounds were dissolved in DMSO and evaluated in 100-μL assay mixtures containing components specific for each enzyme (see below). Reduction of the *trans*-2-octenoyl N-acetylcysteamine (t-o-NAC thioester) substrate analog was measured spectrophotometrically following the utilization of NADH or NADPH at 340 nm at 30°C for the linear period of the assay. *S. aureus* FabI assays contained 50 mM sodium acetate (pH 6.5), 200 μM t-o-NAC thioester, 200 μM NADPH, and 150 nM *S. aureus* FabI.

NADH was used as a cofactor rather than NADPH for the *E. coli* FabI assay. Substrate concentrations used for the Lineweaver–Burk plot were 100, 200, 300, and 400 μM, whereas the concentrations of the cofactor were 100, 200, 400, and 600 μM. The rate of decrease in the amount of NADPH in each reaction was measured with a microtiter enzyme-linked immunosorbent assay (ELISA) reader by using the SOFTmax PRO software (Molecular Devices, Sunnyvale, CA, USA). The inhibitory activity was calculated according to the following formula: % of inhibition  = 100× [1− (rate in the presence of compound/rate in the untreated control)]. IC_50_ values were calculated by fitting the data to a sigmoid equation. An equal volume of DMSO solvent was used for the untreated control. FabK assays contained 100 mM sodium acetate (pH 6.5), 2% glycerol, 200 mM NH_4_Cl, 50 µM t-o-NAC thioester, 200 µM NADH, and 150 nM *S. pneumoniae* FabK.

### Fluorescence quenching assay

Fluorescence spectra were measured using a SHIMADZU fluorescence spectrophotometer (model RF-5310PC). *S. aureus* FabI (15 ng/μl) was incubated with different concentrations of triclosan (1, 2, 4, 8, and 16 nM in PBS buffer) and compounds **1**, **5**, or **7** (10, 20, 40, 80, and 160 nM in PBS buffer). Protein quenching was monitored at 25°C by using 5-nm excitation and 5-nm emission wavelength. The excitation wavelength was 280 nm, and the emission spectra were measured between 290 and 430 nm.

### Determination of minimum inhibitory concentrations (MICs)

Whole-cell antimicrobial activity was determined by broth microdilution as described previously [21]. The test strains except for *S. pneumoniae* were grown to mid-log phase in Mueller–Hinton broth and diluted 1,000-fold in the same medium. Cells (10^5^/mL) were inoculated into Mueller–Hinton broth and dispensed at 0.2 mL/well into a 96-well microtiter plate. *S. pneumoniae* was grown in tryptic soy broth supplemented with 5% sheep blood. MICs were determined in triplicate by serial 2-fold dilutions of test compounds. The MIC was defined as the concentration of a test compound that completely inhibited cell growth during a 24-h incubation at 30°C. Bacterial growth was determined by measuring the absorption at 650 nm by using a microtiter ELISA reader.

### Measurement of the inhibition of macromolecular biosynthesis

To monitor the effects of compound **1** on lipid, DNA, RNA, protein, and cell wall biosynthesis, its effects on the incorporation of [1-^14^C] acetate (50 mCi/mmol), [2-^14^C] thymidine (59.8 mCi/mmol), [U-^14^C] uridine (539 mCi/mmol), L-[U-^14^C] leucine (306 mCi/mmol) or L-[U-^14^C] isoleucine (329 mCi/mmol), and N-acetyl-d-[1-^14^C] glucosamine (58.1 mCi/mmol) into *S. aureus* and *S. pneumoniae* were measured as described previously [21]. *S. aureus* was exponentially grown to an A_650_ of 0.2 in Mueller–Hinton broth. *S. pneumoniae* was grown in tryptic soy broth supplemented with 5% sheep blood. Each 1-mL culture was treated with drugs at 2 times the MIC for 10 min. An equal volume of DMSO solvent was added to the untreated control. After incubation with the radiolabeled precursors at 37°C for 1 h, followed by centrifugation, the cell pellets were washed twice with PBS buffer. After acetate incorporation, the total cellular lipids were extracted with chloroform-methanol-water. The incorporated radioactivity in the chloroform phase was measured by scintillation counting. For the other precursors, incorporation was terminated by adding 10% (w/v) TCA and cooling on ice for 20 min. The precipitated material was collected on Whatman GF/C glass microfiber filters, washed with TCA and ethanol, dried, and counted using a scintillation counter. The total counts incorporated at 1 h of incubation without inhibitors ranged from >7,000 for [U-^14^C] uridine to <13,000 for [1-^14^C] acetate. The inhibition of radiolabeled precursor incorporation was calculated using the following formula: % inhibition  = 100× [1− (radioactivity values of the treated samples/control (no antibacterial) values)]. In all experiments, known antibacterial agents were included as positive controls.

### Frequency of the spontaneously resistant mutant

The frequency of spontaneous resistance was determined for *S. aureus* RN4220, *S. aureus* KCTC 1916, and *E. coli* KCTC 1942. *E. coli* KCTC 1942 is highly sensitive to antibiotics. The organisms were grown to log-phase by dilution of an overnight culture in fresh media and re-incubation at 35°C until the cultures reached a cell density of approximately 10^9^ CFU/mL. A volume of 100 μl of the bacterial suspension was then applied to solid media containing 4× MIC of **1**, **5**, or triclosan. Inocula were determined by applying 100 μl of 10-fold dilutions on solid media without drug. Colony-forming units were counted after 48 h incubation at 35°C. The ratio of the number of colonies on drug-containing plates to that on control plates was calculated as the *in vitro* frequency of isolation of CFU.

### Overexpression assay

An overexpression assay using *S. aureus* RN4220, *S. aureus* RN4220 (pE194), and *S. aureus* RN4220 (pE194-*fabI*) was conducted to perform target validation of FabI inhibitors as described previously [21]. Additionally, both *fabI*- and *fabK*-overexpressing *E. coli* were constructed to test a multitarget effect of the compounds. The wild-type *fabI* gene from the genomic DNA of *E. coli* W3110 was amplified by PCR by using the primers 5′-ATGGGTTTTCTTTCCGGTAAGCGCA-3′ and 5′-TTTCAGTTCGAGTTCGTTCATT-3′. The wild-type *fabK* gene from the genomic DNA of *S. pneumoniae* KCTC 5412 was amplified by PCR by using the primers 5′-ATGAAAACGCGTATTACA-3′ and 5′-GTCATTTCTTAC AACTCCTGTCCA-3′. The resulting products were cloned into the pBAD-TOPO TA expression vector (Invitrogen, Carlsbad, CA, USA) to yield the pBAD-*fabI* and pBAD-*fabK* recombinant plasmids, which placed the expression of the genes *fabI* and *fabK*, respectively, under the control of the arabinose promoter [25]. Recombinant pBAD-*fabI* and pBAD-*fabK* were then introduced into the pump-negative (tolC) *E. coli* EW1b via electroporation to generate *E. coli* EW1b (pBAD-*fabI*) and *E. coli* EW1b (pBAD-*fabK*), respectively.

## Results

### Isolation of meleagrin as a new FabI inhibitor

A FabI inhibitor was isolated from *Penicillium chrysogenum* F717, which is known as a penicillin-producing species. MS and NMR spectral analyses of the inhibitor revealed that it was meleagrin (**1**) (Fig. 1). Compound **1** inhibited both *E. coli* and *S. aureus* FabI with IC_50_ values of 33.2 and 40.1 μM, respectively (Table 1). To determine whether compound **1** selectively inhibited FabI, its effect on FabK, which is the enoyl-ACP reductase of *S. pneumoniae*, was examined. Compound **1** did not inhibit *S. pneumoniae* FabK even at 200 μM, which indicates that it is selective for FabI.

**Table 1:** Comparison of the inhibitory effects of meleagrin (1) and its derivatives against *Staphylococcus aureus* and *E. coli* FabI, bacterial growth, and [^14^C] acetate and [^14^C] leucine incorporation into membrane fatty acids.

Compounds	IC_50_ (μM)			MIC (μg/mL)			IC_50_ (μM)	
	saFabI	ecFabI	spFabK	*S. aureus* a	*E. coli* ^b^	*S. pneumoniae* ^c^	[^14^C] acetate incorporation	[^14^C] leucine incorporation
**1**	40.1	33.2	>200	64	32	64	40.3	>200
**2**	54.6	49.7	>200	64	64	64	-	-
**3**	38.7	38.8	>200	64	32	64	33.5	>200
**4**	48.0	33.1	>200	64	32	64	35.7	>200
**5**	13.5	15.6	>200	16	8	16	16.3	>200
**6**	13.1	15.4	>200	16	8	16	19.8	>200
**7**	>200	>200	>200	>128	>128	>128	>200	>200
Triclosan	0.66	0.98	>200	0.01	0.01	64	0.04	>10

a *S. aureus* RN4220; ^b^ *E. coli* KCTC 1924; ^c^ *S. pneumoniae* KCTC 3932.

### Mode of FabI inhibition

**Figure 2:**
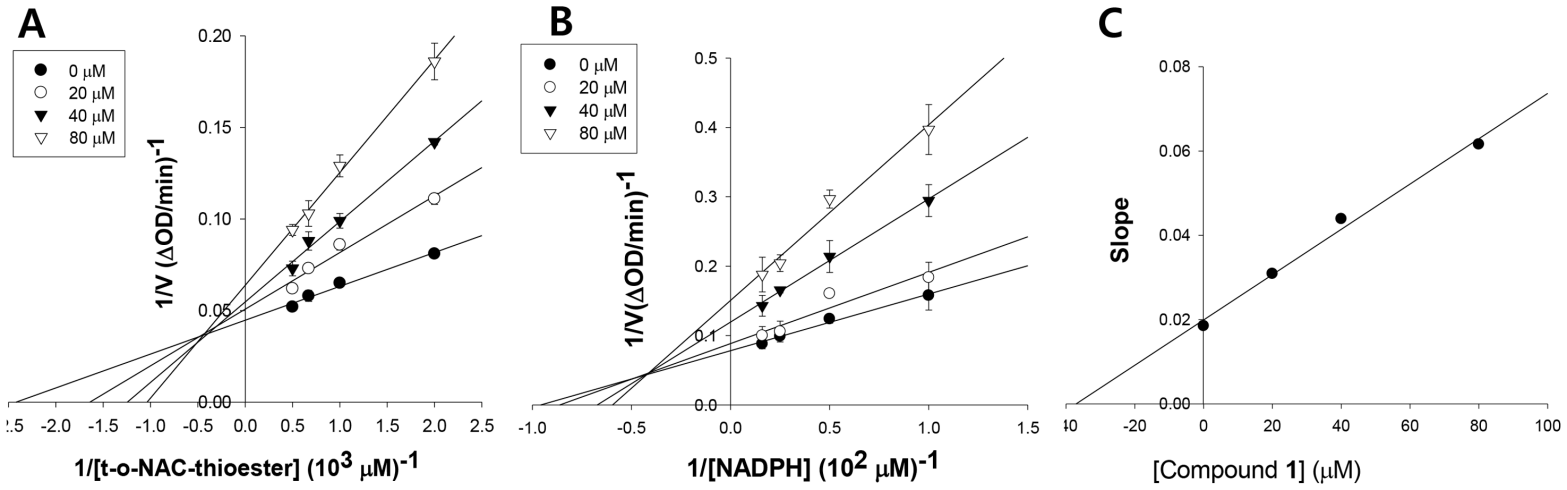
The mechanism of inhibition of *_Staphylococcus aureus_* FabI by meleagrin respective to t-o-NAC thioester (A) and NADPH (B), and *_Ki_* determination of meleagrin (C).

The FabI reaction mechanism requires the nucleotide cofactors NADH or NADPH as the first substrates [26]. The FabI inhibitor could bind to the free enzyme, the enzyme-substrate complex, or both to prevent catalysis. In the first case, the inhibition pattern with respect to the cofactor would be competitive; in the second, the inhibition pattern would be non-competitive; and in the third case, mixed-type inhibition would occur. Inhibition of *S. aureus* FabI by compound **1** was mixed with respect to *trans*-2-octenoyl N-acetylcysteamine, with a *K_i_* value of 39.8 μM (Fig. 2A and 2C). In addition, compound **1** exhibited mixed inhibition with respect to NADPH, with a *K_i_* value of 32.3 μM (Fig. 2B). Thus, compound **1** must bind to both the free enzyme and the FabI-NADPH complex to prevent binding of the nucleotide cofactor and the substrate, respectively.

### Effects of structural changes in compound 1 on FabI and related activity

To determine whether structural changes in compound **1** influence its effects on FabI, compound **1** and its derivatives were tested against *S. aureus* and *E. coli* FabI and bacterial growth (Table 1). Compounds **5** and **6**, which were modified at both the 9-OH and 14-NH groups, produced a significant increase in *S. aureus* and *E. coli* FabI-inhibitory activity, and they enhanced antibacterial activity against *S. aureus* and *E. coli*. In contrast, compounds **2**, **3**, and **4**, which were modified at the 1-NH, 9-OH, and 14-NH groups, respectively, did not affect activity. Compound **7**, which was brominated at the benzene ring of compound **2**, totally lost its activity.

### Effects on fluorescence quenching of *S. aureus* FabI

**Figure 3:**
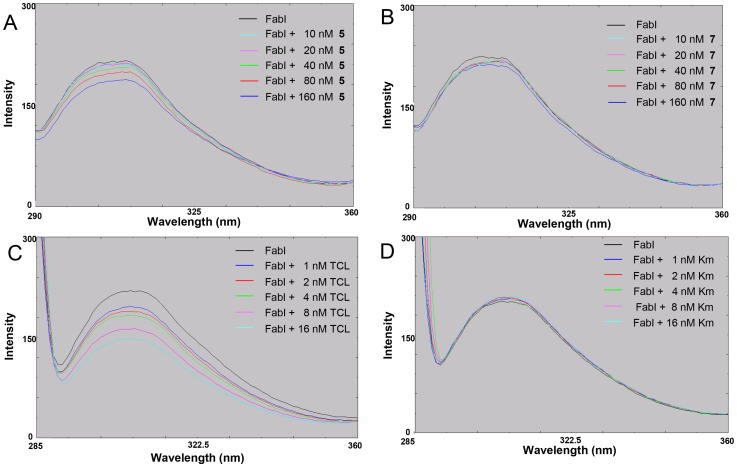
Direct binding of the derivatives of meleagrin with *_Staphylococcus aureus_* FabI by fluorescence quenching assay. (A) The more active derivative (**5**), (B) The inactive derivative (**7**), (C) triclosan (TCL) as a positive control, and (D) kanamycin (Km) as a negative control.

We examined whether active compounds directly bind with FabI by fluorescence quenching analysis. *S. aureus* FabI displayed strong maximal fluorescence at 307 nm after excitation at 270 nm (Fig. 3), whereas triclosan, kanamycin, **5**, and **7** had no fluorescence at this wavelength (data not shown). When *S. aureus* FabI was incubated with increasing amounts of active compound **5**, its fluorescence intensity decreased gradually (Fig. 3A), whereas the inactive compound **7** did not exhibit such an effect (Fig. 3B). Compound **1** showed the same pattern as compound **5** (data not shown). As a positive control, triclosan binding resulted in fluorescence quenching of *S. aureus* FabI (Fig. 3C), whereas kanamycin as a negative control did not (Fig. 3D). These data indicate that the active compounds **1** and **5** directly interact with *S. aureus* FabI, whereas compound **7** does not, thus explaining their effects on FabI.

### Inhibition of cellular fatty acid synthesis

To evaluate whether the active compounds inhibit cellular fatty acid synthesis, we determined whether the compounds inhibited the incorporation of acetate into membrane fatty acids *in vivo*. We measured their effects on the incorporation of [1-^14^C] acetate into membrane fatty acids in *S. aureus*. In agreement with their antibacterial activity and FabI-inhibitory activity, the more active compounds **5** and **6** indeed blocked incorporation of radioactively-labeled acetate into chloroform/methanol-extractable phospholipids *in vivo* in a concentration-dependent manner, with approximately 2-fold higher activity than the less active compounds **1**, **3**, and **4** (Table 1). The inactive compound **7** did not exhibit such fatty acid synthesis inhibition even at 200 μM, as expected. As a positive control, triclosan inhibited fatty acid synthesis in a concentration-dependent manner (data not shown). In contrast, the incorporation of leucine into proteins was not inhibited by the active compounds (Table 1), whereas the protein synthesis inhibitor, chloramphenicol, inhibited incorporation (data not shown).

### Antibacterial activity

Consistent with their FabI-inhibitory activity, compounds **5** and **6** showed 2–4 times higher antibacterial activity than compound **1** against *S. aureus* RN4220 and the highly sensitive strain *E. coli* KCTC 1924 (Table 1), as expected. Interestingly, compounds that were inactive against the FabK isoform exhibited antibacterial activity against *S. pneumoniae* KCTC 3932, which contains only the FabK isoform. This finding suggests that the compounds inhibit not only FabI but also another target. Compounds **5** and **6** also showed antibacterial activity against other gram-positive bacteria, including *S. aureus* 503, *S. aureus* KCTC 1916, MRSA CCARM 3167, MRSA CCARM 3506, QRSA CCARM 3505, QRSA CCARM 3519, *Staphylococcus epidermis* KCTC 3958, *B. subtilis* KCTC 1021, and *Micrococcus luteu*s KCTC 1056 with MIC values of 8–16 μg/mL.

### Effects on *fabI-*overexpressing *S. aureus*

The increase in the MIC for the *fabI*-overexpressing strain relative to the wild type is indicative of FabI being the mode of antibacterial action [27]. The antibacterial activity of the active compounds for the *fabI*-overexpressing strain was investigated to determine whether overexpression of *fabI* shifted the MIC for *S. aureus*. The MICs for the *fabI*-overexpressing strain *S. aureus* RN4220 (pE194-*fabI*) were 4–8-fold higher than those of the wild-type strain *S. aureus* RN4220, or the vector-containing strain *S. aureus* RN4220 (pE194) (Table 2). The MIC for triclosan in the *fabI*-overexpressing strain increased, which was used as a positive control. Erythromycin, the selection marker for the vector pE194, increased the MICs for both the *fabI*-overexpressing strain and the vector-containing strain, which indicated that the engineered constructs functioned as expected. Antibiotics with different modes of action such as oxacillin and norfloxacin were applied as negative controls and did not change the MICs of the 3 strains, which indicates that altered expression of *fabI* does not alter the sensitivity of cells to antibiotics in general. These results indicate that the active compounds inhibited the growth of *S. aureus* by inhibiting the *fabI*-encoded ENR.

**Table 2:** Reduced susceptibility of *fabI*-overexpressing *Staphylococcus aureus* to meleagrin (1) and its derivatives.

Compounds	IC_50_ (μM)	MIC (μg/mL)			Mode of action
	saFabI	Wild type	*S. aureus* (pE194)	*S. aureus* (pE194-fabI)	
**1**	40.1	64	64	256	FabI
**3**	38.7	64	64	256	FabI
**4**	48.0	64	64	256	FabI
**5**	13.5	16	16	128	FabI
**6**	13.1	16	16	128	FabI
Triclosan	0.6	0.01	0.01	1.6	FabI
Erythromycin	>100	0.5	64	64	Protein synthesis
Oxacillin	>100	0.25	0.25	0.25	Cell wall
Norfloxacin	>100	1	1	1	DNA synthesis

### Frequency of spontaneously resistant mutants

We isolated resistant mutants to determine which other gene or genes were targeted by the active compounds (Table 3). As a control, triclosan-resistant mutants were isolated at a frequency of 3.30±0.13×10^−8^, 2.58±0.04×10^−9^, and 9.07±0.08×10^−8^ from *S. aureus* RN4220, *S. aureus* KCTC 1916, and the antibiotic-sensitive *E. coli* KCTC 1942, respectively. However, no mutants resistant to compounds **1** and **5** were detected from the strains tested. These results suggest that compounds **1** and **5** inhibit multiple targets.

**Table 3:** Frequency of resistance to meleagrin (1) and its more active derivative.

Strains	MIC (μg/mL)			Exposure	Frequency of resistance		
	1	5	Triclosan		1	5	Triclosan
*S. aureus* RN4220	64	16	0.01	4× MIC	<1.62×10^−10^	<1.62×10^−10^	3.30±0.13×10^−8^
*S. aureus* KCTC 1916	32	16	0.01	4× MIC	<1.03×10^−10^	<1.03×10^−10^	2.58±0.04×10^−9^
*E. coli* KCTC 1924	32	8	0.02	4× MIC	<6.7×10^−9^	<6.7×10^−9^	9.07±0.08×10^−8^

### Effects on macromolecular biosynthesis

To identify other pathways inhibited by compound **1**, the effects of compound **1** on the incorporation of radiolabeled precursors of macromolecular synthesis in *S. pneumoniae* and in *S. aureus* were investigated. All reference antibacterial agents selectively inhibited the macromolecular synthesis pathway, which is consistent with their known mechanism of action (Table 4). Compound **1** inhibited the incorporation of acetate into lipids in both *S. aureus* and *S. pneumoniae* by 62% and 65%, respectively, whereas the incorporation of thymidine, uridine, isoleucine, and N-acetylglucosamine, into DNA, RNA, protein, and the cell wall, respectively, was not inhibited. Because compound **1** is inactive against the FabK isoform, these data suggest that compound **1** inhibits at least one additional target in addition to FabI in the fatty acid pathway.

**Table 4:** Effects of meleagrin (1) on incorporation of radiolabeled precursors into *S. aureus* and *S. pneumoniae*.

Strains	Compounds	Inhibition of precursor incorporation (%)				
		[1-^14^C] Acetate	[2-^14^C] Thymidine	[U-^14^C] Uridine	L-[U-^14^C] Isoleucine	N-Acetyl-D-[1-^14^C] Glucosamine
*S. aureus* a	Reference antibacterial^c^	87	79	69	74	79
	**1**	62	13	17	6	25
*S. pneumoniae* ^b^	Reference antibacterial^d^	95	83	92	85	88
	**1**	65	15	2	9	3

a *S. aureus* RN4220; ^b^ *S. pneumoniae* KCTC 3932. ^c^Reference antibacterials used for inhibition of acetate, thymidine, uridine, isoleucine, and N-acetyl-d-glucosamine incorporation are triclosan, norfloxacin, rifampin, chlorampenicol, and vancomycin, respectively. ^d^Reference antibacterials in *S. pneumoniae* were the same as in *S. aureus*, except cerulenin was used instead of triclosan for acetate inhibition.

### Effects on *fabK*-overexpressing *E. coli*

To demonstrate that active compounds **1** and **5** inhibit not only FabI but also an additional target, we cloned *fabK* and *fabI* into an arabinose-inducible expression system, vector pBAD TOPO, and placed this plasmid in a TolC-negative *E. coli* host. Because FabK is resistant to compounds **1** and **5**, if the compounds inhibited only FabI, expression of FabK in *E. coli* would lead to resistance to compounds **1** and **5** because the expressed FabK can compensate for the inhibited FabI. As expected, the MICs of compounds **1** and **5** for *fabI*-overexpressing *E. coli* EW1b (pBAD-fabI) were 4-fold higher than those for wild-type *E. coli* EW1b and vector-containing *E. coli* EW1b (pBAD) in the presence of arabinose (Table 5). However, the MICs for the *fabK*-overexpressing *E. coli* EW1b (pBAD-fabK) did not change. As a positive control, triclosan, which does not inhibit FabK, showed inducer-dependent higher MICs for *fabK*-overexpressing *E. coli* and *fabI*-overexpressing *E. coli*. Therefore, *S. pneumoniae* FabK replaced *E. coli* FabI for fatty acid synthesis, which, in turn, indicates that FabI is the only target of triclosan in this system. Ampicillin, which is the selection marker for the pBAD vector, increased the MICs for all vector-containing strains, thereby demonstrating normal functioning of the constructs. Actinonin, which is a PDF inhibitor applied as a negative control, did not change the MICs of any of the tested strains. This result clearly indicates that active compounds **1** and **5** inhibit an additional target as well as FabI, unlike triclosan.

**Table 5:** Unchanged susceptibility of *fabK*-overexpressing *E. coli* to meleagrin (1) and its derivative (MIC, μg/mL).

Compounds	*E. coli* EW1b		*E. coli* EW1b(pBAD)		*E. coli* EW1b(pBAD-*fabI*)		*E. coli* EW1b(pBAD-*fabK*)	
	(−) Ara	(+) Ara^a^	(−) Ara	(+) Ara	(−) Ara	(+) Ara	(−) Ara	(+) Ara
**1**	32	32	32	32	32	128	32	32
**5**	16	16	16	16	16	64	16	16
Triclosan	0.002	0.002	0.002	0.002	0.002	>0.08	0.002	>0.08
Ampicillin	1	1	>125	>125	>125	>125	>125	>125
Actinonin	1	1	1	1	1	1	1	1

a 3% arabinose was treated.

## Discussion

We screened 25,000 microbial extracts consisting of actinomycetes and fungi to identify new FabI inhibitors. Meleagrin was isolated from the solid-state fermentation of the fungal strain *P. chrysogenum* F717. Meleagrin was previously isolated from *P. meleagrinum* [28] and *P. chrysogenum* [29], but its biological activity, including antimicrobial activity, has not been reported. Although its activity was weak, meleagrin clearly showed inhibition selective for *S. aureus* FabI over *S. pneumoniae* FabK. Importantly, the binding of meleagrin with *S. aureus* FabI was demonstrated by the fluorescence quenching assay. Furthermore, its inhibition of FabI was supported by results obtained using its chemical derivatives, the intracellular fatty acid synthesis assay, and the *fabI*-overexpressing assay. Interestingly, meleagrin and its more active derivatives showed antibacterial activity against *S. pneumoniae*, in which FabK is the sole enoyl-ACP reductase, and it did not produce spontaneously resistant mutants of *S. aureus* or *E. coli*, in contrast to triclosan, which suggests that meleagrin inhibits multiple targets. Meleagrin inhibited the incorporation of radiolabeled acetate into lipids in *S. pneumoniae* and *S. aureus*, whereas incorporation of thymidine (DNA), uridine (RNA), isoleucine (protein), and N-acetylglucosamine (cell wall) was not inhibited, which indicates that these compounds inhibit fatty acid synthesis through one or more modes of action in addition to FabI inhibition. The multitarget effect was confirmed by the *fabK*-overexpression assay in *E. coli*. The multitarget effect is very important from the point of view of drug development because a single point mutation in one gene for a drug with a single target renders the strain resistant and the drug useless. Thus, when considering that one of the advantages of antibacterial agents having multiple targets is the reduced development of drug resistance [10], meleagrin and its derivatives hold promise for the development of new antibiotics that can treat infections caused by multidrug-resistant pathogens.

Several FabI inhibitors have been reported, and most were derived from compound libraries and were synthetically developed using structure-based approaches, including 1,4-disubstituted imidazoles, aminopyridines, naphthyridinones, and thiopyridines [30]. Although synthetic inhibitors are potent, they have a disadvantage, as resistant mutants occur at relatively high frequency [16], A few natural FabI inhibitors have been reported, such as vinaxanthone [21], cephalochromin [31], kalimantacin/batumin [22], EGCG, and flavonoids [32]. EGCG and flavonoids inhibit several targets such as FabG, FabZ, and FabI. The mode of action of vinaxanthone, cephalochromin, and kalimantacin/batumin was demonstrated by FabI-overexpressing strains. To our knowledge, this is the first study on a multitarget effect of FabI inhibitors.

In summary, meleagrin is a new class of FabI inhibitor with antibacterial activity against multidrug-resistant bacteria such as MRSA and QRSA. Meleagrin is structurally unique, and it inhibits at least one more target in addition to FabI, thereby resulting in a no resistance mutant; thus, meleagrin may have potential as a useful lead compound for the development of a new anti-MRSA agent.

## Supporting Information

Information S1 **Preparation and spectral data of compounds 2–7.**
(DOCX)
