# Supplementary material for: Meleagrin, a New FabI Inhibitor from Penicillium chryosogenum with at Least One Additional Mode of Action
Source: PLoS One. 2013 Nov 28;8(11):e78922. doi: 10.1371/journal.pone.0078922 (PMC3842914; doi:10.1371/journal.pone.0078922)
Supplement: Information S1 — Preparation and spectral data of compounds 2–7. (DOCX) [file pone.0078922.s001.docx]

**Supporting Information**

**The preparation methods and spectral data of compounds 2 – 7**.

*Demethoxylation of compound* **1** - Compound **1** was subjected to demethoxylated reaction conditions by treating a solution of compound **1** (20 mg) in CH_2_Cl_2_ (4 mL) with 1 M BBr_3_ solution in CH_2_Cl_2_ (0.3 mL) at 0°C for 3 h. The reaction products were purified over a reverse phase HPLC column (20 × 150 mm, YMC C18) and eluted with MeOH: H2O (70:30) at a flow rate of 4.5 mL/min, which yielded compounds **2** and **7** at retention times of 33.9 and 35.1, min, respectively. Compound **2** was determined to be a *N*-demethoxy compound, which has been reported as glandicolin **A** [[1](#_ENREF_1)], by the following MS and NMR spectral data; ESI-MS: *m/z* 402.6 (M-H)^-^, 404.6 (M+H)^+^, 426.5 (M+Na)^+^, C_22_H_21_N_5_O_3_ requires 403.16; ^1^H-NMR (300 MHz, CD_3_OD): 8.23 (1H, s, H-15), 7.81 (1H, s, H-20), 7.51 (1H, d, *J* = 7.6, H-4), 7.32 (1H, s, H-18), 7.25 (1H, t, *J* = 7.6, H-6), 7.03 (1H, t, *J* = 7.6, H-5), 6.67 (1H, d, *J* = 7.6, H-7), 6.08 (1H, brs, H-22), 5.33 (1H, s, H-8), 5.04 (1H, d, *J* = 17.7, Ha-23), 4.95 (1H, d, *J* = 11.1, Hb-23), 1.28 (6H, s, CH_3_-24 and 25). Compound **7** was determined to be a new derivative of glandicolin A brominated at C-7 based on the following MS and NMR spectral data; [α]_D_ = -149.5° (*c* = 0.04, MeOH); ESI-MS: *m/z* 480.5 (M-H)^-^, 482.5 [(M+2)-H]^-^, 482.5 (M+H)^+^, 484.5 [(M+2)+H]^+^, C_22_H_20_BrN_5_O_3_ requires 481.07; ^1^H-NMR (500 MHz, CD_3_OD): 8.26 (1H, s, H-15), 7.77 (1H, s, H-20), 7.50 (1H, dd, *J* = 7.5 and 1.0, H-4), 7.34 (1H, s, H-18), 7.26 (1H, dd, *J* = 7.5 and 1.0, H-6), 6.76 (1H, t, *J* = 7.5, H-5), 6.09 (1H, brs, H-22), 5.32 (1H, s, H-8), 5.06 (1H, d, *J* = 17.0, Ha-23), 4.98 (1H, d, *J* = 9.0, Hb-23), 1.29 (6H, s, CH_3_-24 and 25).

*Methylation of compound* ***1*** - Treatment of compound **1** (20 mg) with ethereal diazomethane (2.0 mmol) at room temperature and purification by SiO_2_ thin layer chromatography developed with CHCl_3_:MeOH = 15:1 yielded methylated compounds **3**, **4**, and **5** at *R_f_* values of 0.3, 0.25, and 0.4, respectively. Compound **3** was determined to be *O*-methylmeleagrin, which is known as oxaline,[[2](#_ENREF_2)] based on the following MS and NMR spectral data; ESI-MS: *m/z* 446.5 (M-H)^-^, 448.5 (M+H)^+^, C_24_H_25_N_5_O_4_ requires 447.19; ^1^H-NMR (300 MHz, CDCl_3_): 8.42 (1H, s, H-15), 7.67 (1H, s, H-20), 7.59 (1H, d, *J* = 7.8, H-4), 7.34 (1H, s, H-18), 7.30 (1H, t, *J* = 7.8, H-6), 7.09 (1H, t, *J* = 7.6, H-5), 6.68 (1H, d, *J* = 7.8, H-7), 6.15 (1H, brs, H-22), 5.13 (1H, s, H-8), 5.10 (1H, d, *J* = 17.1, Ha-23), 5.05(1H, d, *J* = 10.0, Hb-23), 3.76 (3H, s, 1-OCH_3_), 3.65 (3H, s, 9-OCH_3_), 1.26 (6H, s, CH_3_-24 and 25). Compound **4** was determined to be *N^14^*-methylmeleagrin [[2](#_ENREF_2)] based on the following MS and NMR spectral data; ESI-MS: *m/z* 446.5 (M-H)^-^, 448.5 (M+H)^+^, C_24_H_25_N_5_O_4_ requires 447.19; ^1^H-NMR (300 MHz, CDCl_3_): 8.30 (1H, s, H-15), 7.71 (1H, s, H-20), 7.54 (1H, d, *J* = 7.8, H-4), 7.38 (1H, s, H-18), 7.32 (1H, t, *J* = 7.8, H-6), 7.05 (1H, t, *J* = 7.6, H-5), 6.91 (1H, d, *J* = 7.8, H-7), 6.15 (1H, dd, *J* = 17.7 and 10.8, H-22), 5.45 (1H, s, H-8), 5.05 (1H, d, *J* = 17.1, Ha-23), 5.02 (1H, d, *J* = 10.8, Hb-23), 3.74 (3H, s, 1-OCH_3_), 2.46 (3H, s, 14-CH_3_), 1.26 and 1.21 (3H, each, s, CH_3_-24 and 25). Compound **6** was determined to be *O,N^14^*-dimethylmeleagrin [[2](#_ENREF_2)] based on the following MS and NMR spectral data; ESI-MS: *m/z* 460.6 (M-H)^-^, 462.6 (M+H)^+^, C_25_H_27_N_5_O_4_ requires 461.21; ^1^H-NMR (300 MHz, CDCl_3_): 8.45 (1H, s, H-15), 7.69 (1H, s, H-20), 7.56 (1H, d, *J* = 7.7, H-4), 7.37 (1H, s, H-18), 7.29 (1H, t, *J* = 7.7, H-6), 7.06 (1H, t, *J* = 7.7, H-5), 6.93 (1H, d, *J* = 7.7, H-7), 6.12 (1H, dd, *J* = 17.7 and 11.1, H-22), 5.09 (1H, s, H-8), 5.05 (1H, d, *J* = 17.7, Ha-23), 5.03 (1H, d, *J* = 11.1, Hb-23), 3.76 (3H, s, 1-OCH_3_), 3.62 (3H, s, 9-OCH_3_), 2.44 (3H, s, 14-CH_3_), 1.31 and 1.21 (3H, each, s, CH_3_-24 and 25).

*Preparation of compound* ***6 –*** Compound **6** was prepared by treating compound **2** with diazomethane at room temperature. Compound **6** was determined to be a new *O, N^14^*-dimethylated derivative of glandicolin A based on the following MS and NMR spectral data; HRESI-MS: *m/z* 432.20236 (M+H)^+^, C_24_H_26_N_5_O_3_ requires 432.20302; ^1^H-NMR (500 MHz, CDCl_3_): 8.35 (1H, s, H-15), 7.64 (1H, s, H-18), 7.31 (1H, s, H-20), 7.18 (1H, t, *J* = 7.5, H-6), 6.92 (1H, t, *J* = 7.5, H-5), 6.72 (1H, d, *J* = 7.5, H-7), 6.16 (1H, dd, J = 17.4 and 10.8, H-22), 5.05 (1H, s, H-8), 5.03 (1H, d, *J* = 10.8, Ha-23), 5.02 (1H, d, *J* = 17.4, Hb-23), 3.59 (3H, s, 9-OCH_3_), 2.65 (3H, s, 14-CH_3_), 1.43 and 1.22 (3H, each, s, CH_3_-24 and 25), ^13^C-NMR (125 MHz, CDCl_3_): 164.3 (C-13), 158.5 (C-10), 146.6 (C-7a), 145.8 (C-9), 143.1 (C-22), 136.9 (C-18), 135.1 (C-20), 128.8 (C-6), 127.1 (C-3a), 126.4 (C-16), 124.3 (C-4), 122.7 (C-12), 120.7 (C-5), 113.5 (C-23), 109.8 (C-15), 109.1 (C-7), 108.4 (C-8), 99.5 (C-2), 56.6 (C-3), 55.8 (9-OCH_3_), 41.2 (C-21), 30.3 (14-CH_3_), 25.1 and 23.3 (C-24 and 25).

**References**

1. Overy DP, Nielsen KF, Smedsgaard J (2005) Roquefortine/oxaline biosynthesis pathway metabolites in Penicillium ser. Corymbifera: in planta production and implications for competitive fitness. J Chem Ecol 31: 2373-2390.

2. Konda Y, Onda M, Hirano A, Omura S (1980) Oxaline and neoxaline. Chem Pharm Bull 28: 2987-2993.
